# Supplementary figures and images for: Probiotics-induced changes in gut microbial composition and its effects on cognitive performance after stress: exploratory analyses
Source: Transl Psychiatry. 2021 May 20;11:300. doi: 10.1038/s41398-021-01404-9 (PMC8137885; doi:10.1038/s41398-021-01404-9)

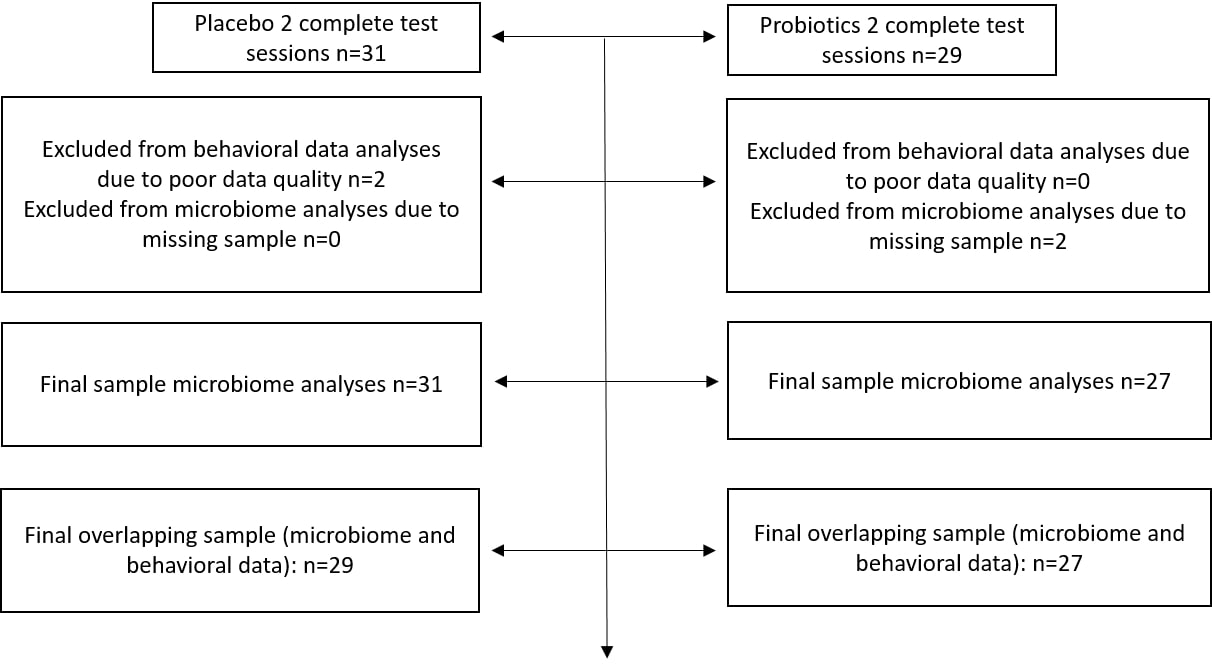

Supplement: Supplementary file 2 — Supplementary Figure 1 [file 41398_2021_1404_MOESM2_ESM.jpg]

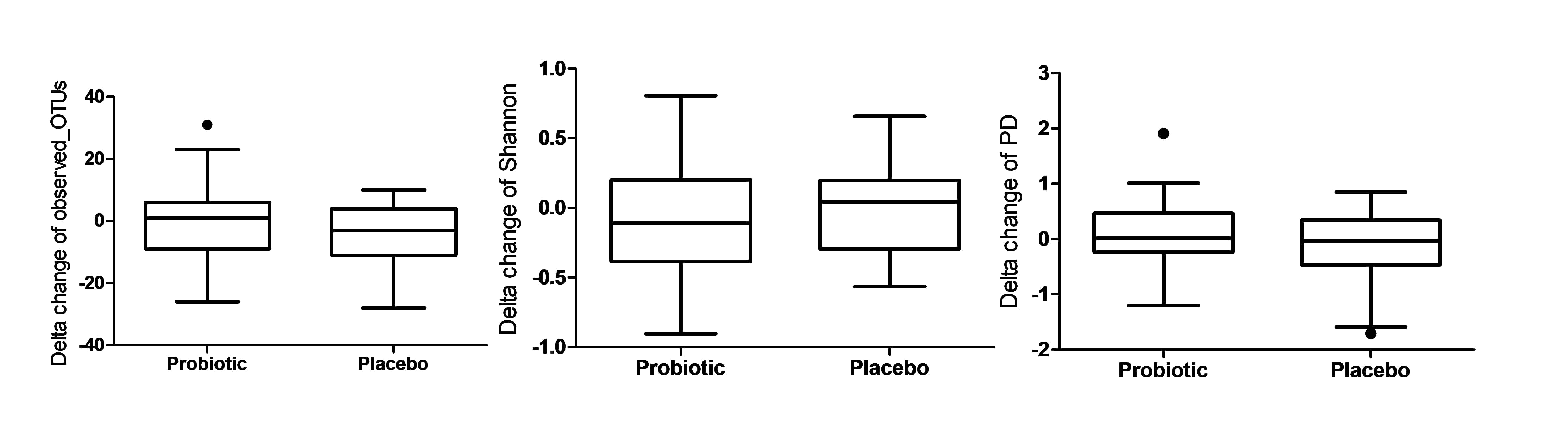

Supplement: Supplementary file 3 — Supplementary Figure 2 [file 41398_2021_1404_MOESM3_ESM.jpg]

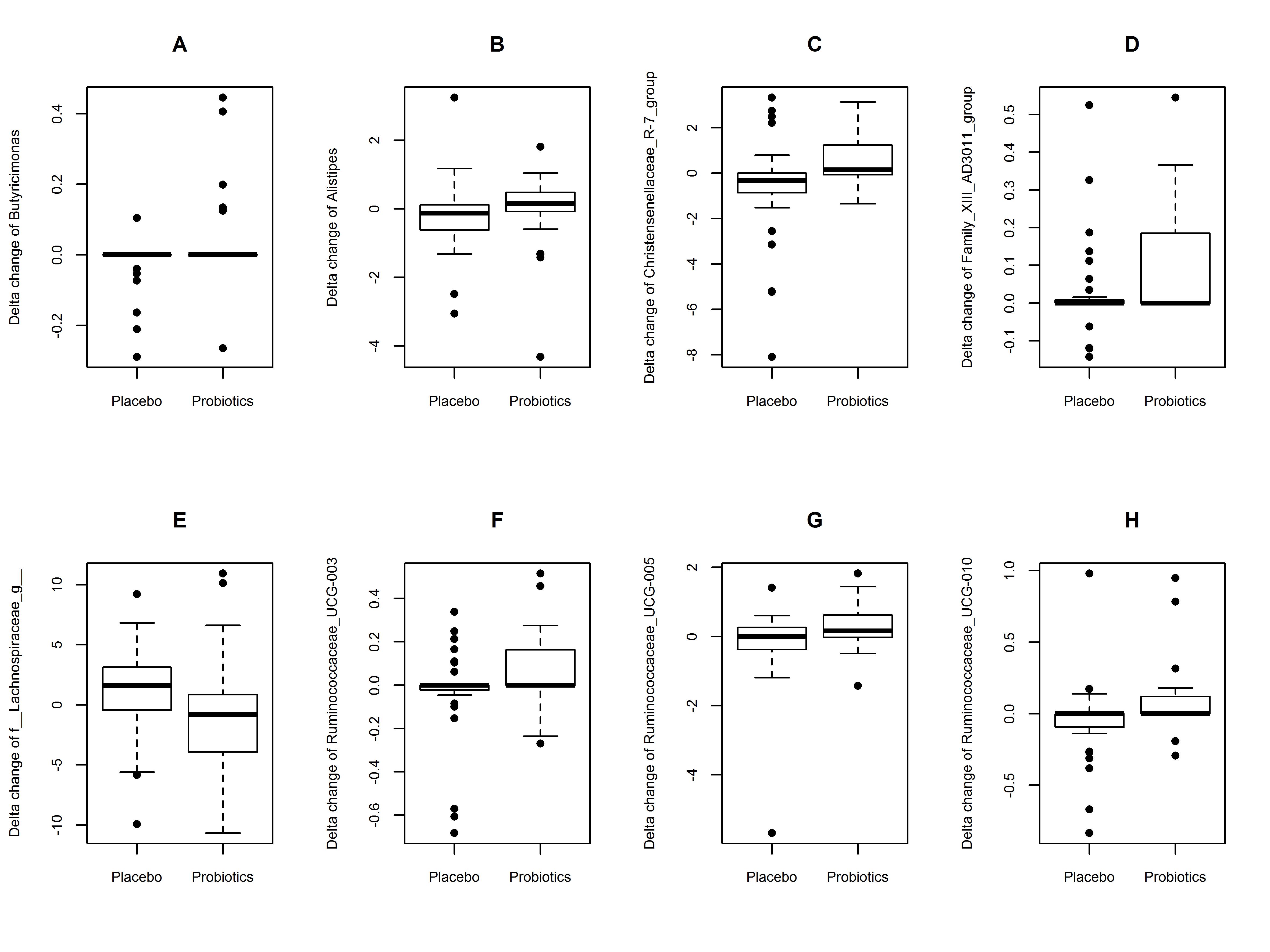

Supplement: Supplementary file 4 — Supplementary Figure 3 [file 41398_2021_1404_MOESM4_ESM.jpg]

A

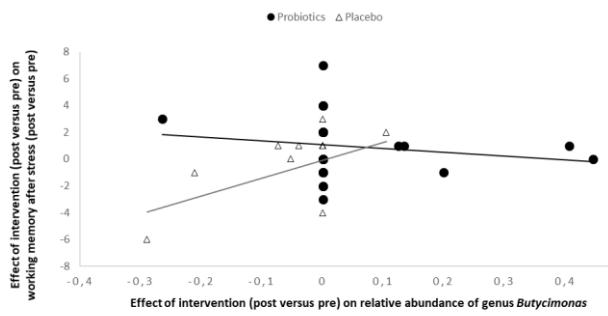

B

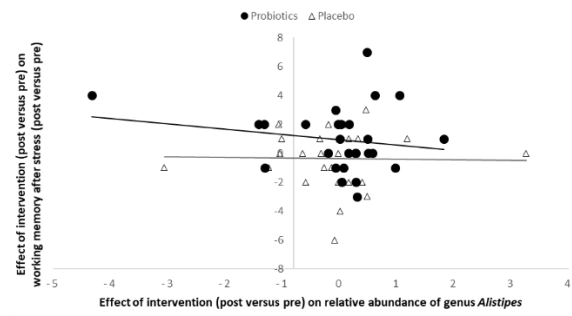

C

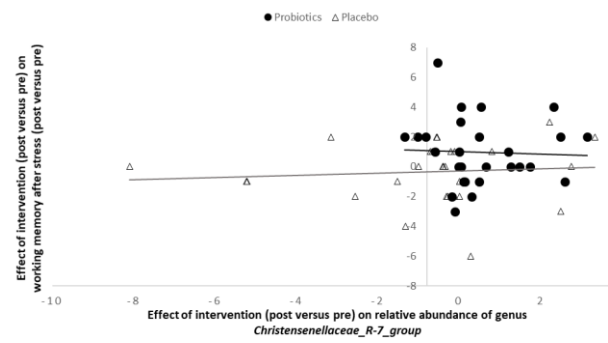

D

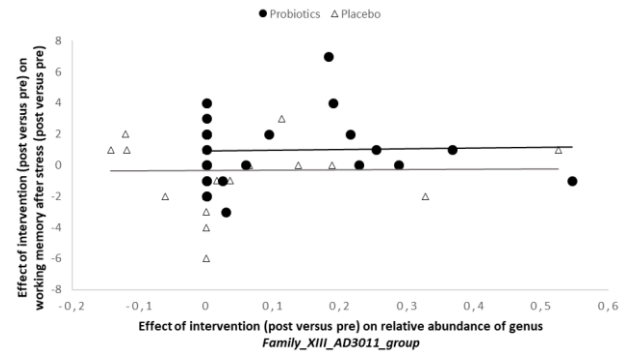

E

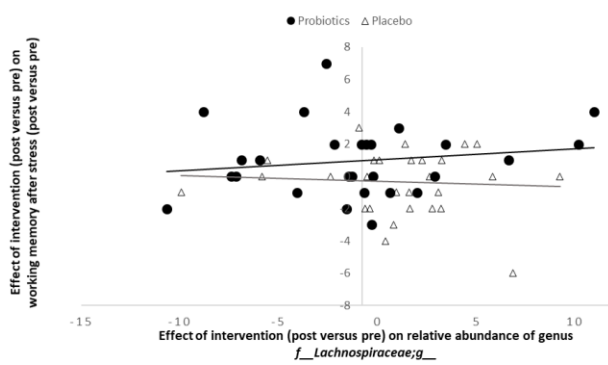

F

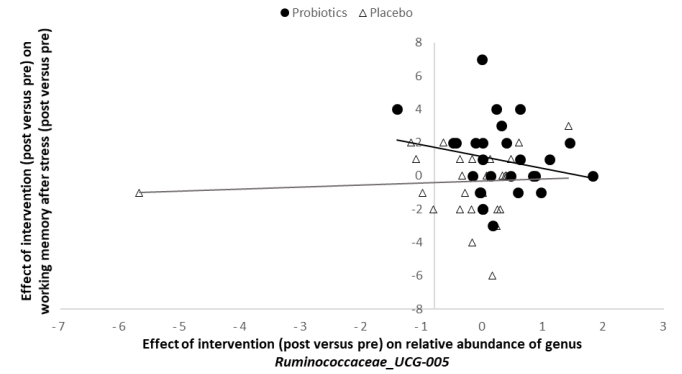

G

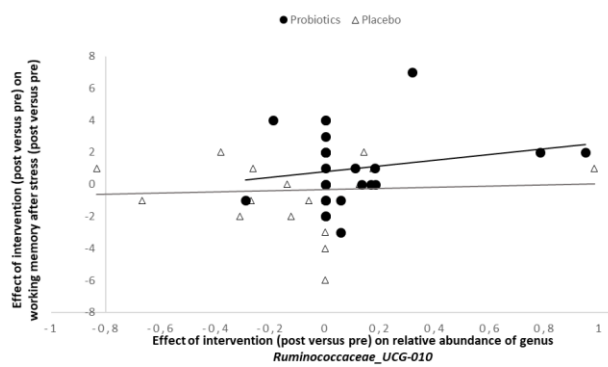

Supplement: Supplementary file 5 — Supplementary Figure 4 [file 41398_2021_1404_MOESM5_ESM.pdf]
